# Field-controllable Spin-Hall Effect of Light in Optical Crystals: A Conoscopic Mueller Matrix Analysis

**DOI:** 10.1038/s41598-018-20402-4

**Published:** 2018-01-31

**Authors:** C. T. Samlan, Nirmal K. Viswanathan

**Affiliations:** School of Physics, University of Hyderabad, Hyderabad, 500046 India

## Abstract

Electric-field applied perpendicular to the direction of propagation of paraxial beam through an optical crystal dynamically modifies the spin-orbit interaction (SOI), leading to the demonstration of controllable spin-Hall effect of light (SHEL). The electro- and piezo-optic effects of the crystal modifies the radially symmetric spatial variation in the fast-axis orientation of the crystal, resulting in a complex pattern with different topologies due to the symmetry-breaking effect of the applied field. This introduces spatially-varying Pancharatnam-Berry type geometric phase on to the paraxial beam of light, leading to the observation of SHEL in addition to the spin-to-vortex conversion. A wave-vector resolved conoscopic Mueller matrix measurement and analysis provides a first glimpse of the SHEL in the biaxial crystal, identified via the appearance of weak circular birefringence. The emergence of field-controllable fast-axis orientation of the crystal and the resulting SHEL provides a new degree of freedom for affecting and controlling the spin and orbital angular momentum of photons to unravel the rich underlying physics of optical crystals and aid in the development of active photonic spin-Hall devices.

## Introduction

The collective and significant role played by the amplitude, phase and polarization of an electromagnetic wave in enriching the fundamental understanding of light field and quanta (photons), is not more evident than in the *spin-orbit interaction* (SOI) of light – a study of the mutual influence of the *spin angular momentum* (SAM) and the intrinsic and extrinsic *orbital angular momentum* (OAM) of light^1^. The concept of SOI of light first proposed and demonstrated by Zel′dovich *et al*.^2^, continues to be a research topic of significant importance, as evinced in the authoritative and comprehensive recent review articles^3–5^. Broadly, the SOI effect results in the spin-dependent redistribution of light intensity due to space- or wavevector-variant geometric phase. In systems with rotational symmetry, the SOI leads to *spin-to-orbital angular momentum conversion* (SOC) and the generation of optical vortex beams; breaking of which results in the *spin-Hall Effect of light* (SHEL), a spin-dependent redistribution of light intensity in the transverse plane.

Of the innumerable effects and devices enabled by the SOI phenomenon, the direct manifestation of SHEL is reported in a verity of systems such as planar dielectric interface^6^, smoothly inhomogeneous medium^2,7^ and metasurfaces^8^, and reviewed recently in ref.^9^, which is of interest to us here. The observation of SHEL is attributed to two ‘seemingly’ different mechanisms: the geometric phase gradient arising from spin-redirection (*Rytov-Vladimirskii-Berry*, RVB) phase, related to variation in the direction of propagation of a paraxial light beam due to medium inhomogeneity and the *Pancharatnam-Berry* (PB) phase associated with the manipulation of the state-of-polarization (SoP) of light via varying fast axis orientation^8–10^. The gradient in RVB and PB phases along any linear direction respectively, in the momentum space and real space manifest themselves as SHEL in the real and momentum space and have been investigated independently in a variety of systems^6–16^. The interplay between the SAM and the extrinsic OAM (trajectory) of paraxial light beam resulting in the SHEL is typically studied by invoking symmetry breaking^11–16^. Though the RVB phase induced SHEL has been widely investigated in reflection and transmission at a refractive index boundary^6,11–13^, the first observation of its PB phase counterpart arising due to paraxial light propagation through a tilted anisotropic wave plate was only recently reported by us^16^. Accordingly, breaking of the rotation symmetry of a c-cut uniaxial crystal due to the application of electric field across it provides an ideal system for investigating the PB phase induced controllable SHEL. The problem in hand can be mathematically quite challenging despite the development of a new formalism based on optical singularities^17^. Nevertheless, the demonstration of important features of the treatment for transparent and chiral crystal are evident from our experimental results. Which also provides answer to the non-conservation of *total angular momentum* (TAM) of the electromagnetic field propagating at an angle with reference to the symmetry-axis of an optical crystal^18,19^. Standard experimental techniques of Stokes polarimetry and quantum weak measurement^6,16^ not readily suitable for measuring the weak SHEL due to the complex PB phase gradient across the beam cross-section due to spatially-varying complex fast axis orientation of the crystal. We develop and demonstrate a wavevector resolved *conoscopic Mueller matrix analysis* (CMMA) to map the complex topological pattern of the crystal fast-axis orientation and its transformation and the resulting appearance of SHEL by measuring the circular birefringence as its manifestation. The effect observed varies across the interaction region, and is enhanced due to the applied electric field, which demonstrates the field-controllable nature of the SHEL. The results are of significance due to emerging interest in the control and tunability of SHEL via different mechanisms^15,20–22^.

**Figure 1:**
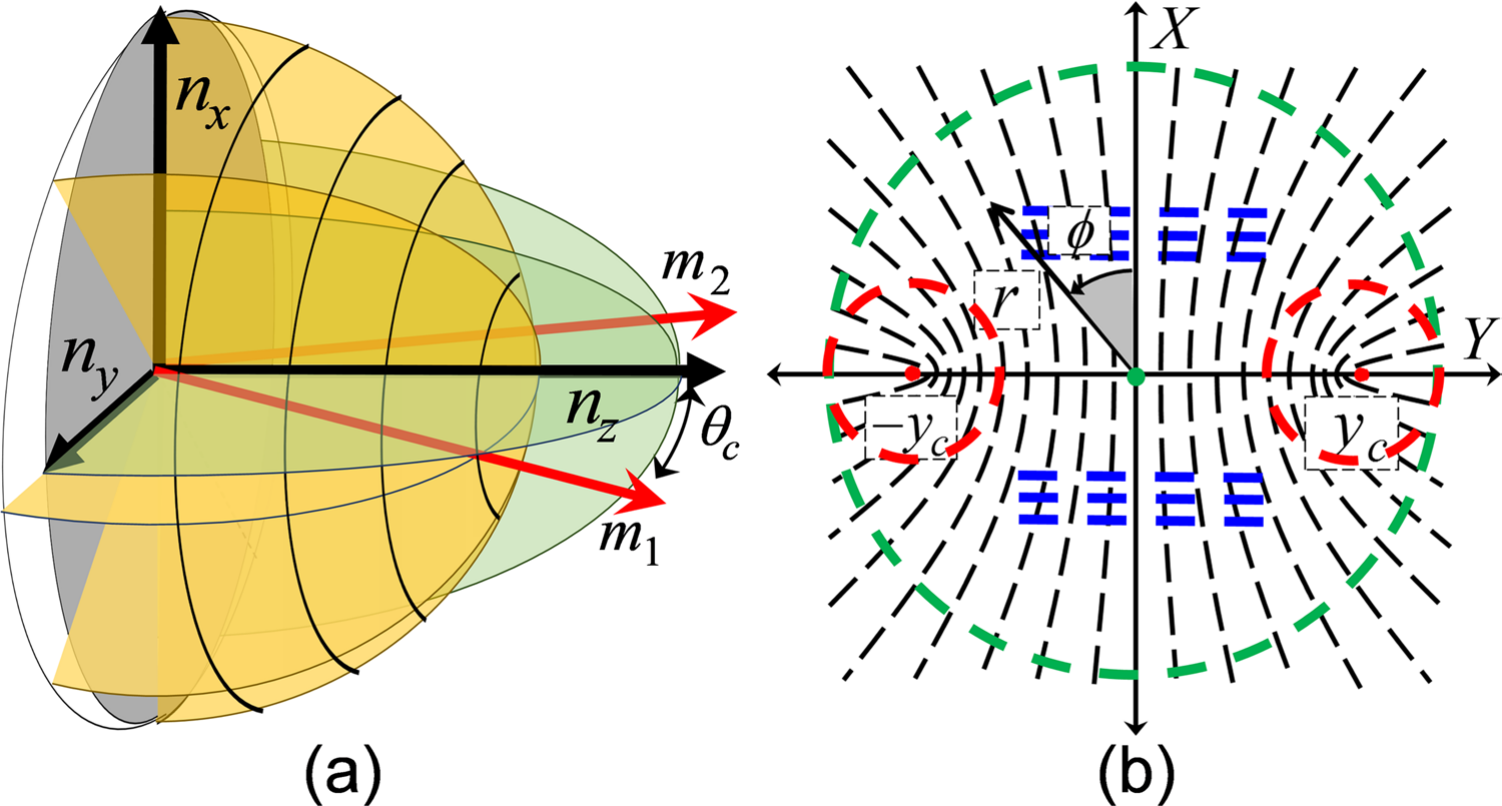
(**a**) Index ellipsoid of a biaxial crystal and (**b**) its fast-axes orientation in the stereographic projection shows different topologies.

To experimentally investigate the field-controlled SHEL, we use Potassium Dihydrogen Phosphate (KDP) crystal, an important class of hydrogen-bonded ferroelectric crystal and one of the most widely investigated optoelectronic material for its linear and nonlinear optical effects and their applications^23–25^. For KDP crystal, non-centrosymmetric, with tetragonal structure (point group $\bar{4}2m$) and 4-fold inversion symmetry are not the only possibility^26,27^. Being an electro-optic as well as a piezo-optic medium, the radially symmetric fast-axis orientation of the uniaxial structure gets modified considerably with the application of external electric field in transverse direction to the propagation of light. The non-vanishing electro-optic coefficient (*r*_41_), piezo-electric constant (*d*_14_) and strain-optic constant (*p*_55_) of the KDP crystal all together contribute to the changes in the fast axis direction as well as the principal refractive index values^26–32^. The corresponding transformation in the index ellipsoid of the KDP crystal from uniaxial tetragonal to biaxial orthorhombic and to biaxial monoclinic can be expressed as,

1 $$\begin{array}{c}(\begin{array}{ccc}{n}_{x} & 0 & 0\\ 0 & {n}_{y} & 0\\ 0 & 0 & {n}_{z}\end{array})=(\begin{array}{ccc}{n}_{{\rm{o}}} & 0 & 0\\ 0 & {n}_{{\rm{o}}} & 0\\ 0 & 0 & {n}_{e}\end{array})\\ \quad \quad \quad \quad \quad \Rightarrow (\begin{array}{ccc}{n}_{{\rm{o}}} & 0 & 0\\ 0 & {n}_{{\rm{o}}}+{n}_{{\rm{o}}}^{3}{E}_{x}^{2}\eta  & 0\\ 0 & 0 & {n}_{{\rm{e}}}-{n}_{{\rm{e}}}^{3}{E}_{x}^{2}\eta \end{array})\\ \quad \quad \quad \quad \quad \Rightarrow (\begin{array}{ccc}{n}_{{\rm{o}}} & 0 & 1/\sqrt{{p}_{55}{d}_{14}{E}_{x}}\\ 0 & {n}_{{\rm{o}}}+{n}_{{\rm{o}}}^{3}{E}_{x}^{2}\eta  & 0\\ 1/\sqrt{{p}_{55}{d}_{14}{E}_{x}} & 0 & {n}_{{\rm{e}}}-{n}_{{\rm{e}}}^{3}{E}_{x}^{2}\eta \end{array})\end{array}$$

where, $\eta =\frac{{r}_{41}^{2}}{2}\frac{{n}_{{\rm{o}}}^{2}{n}_{{\rm{e}}}^{2}}{{n}_{{\rm{o}}}^{2}-{n}_{{\rm{e}}}^{2}}$ and the applied electric field is along the *x*-axis (*E*_*x*_). The off-diagonal elements in the last matrix can be assumed to be negligible for lower values of *E*_*x*_. Thus, under the influence of external field, the fast axis orientation of the KDP crystal gets modified significantly from radial symmetry as shown in Fig. 1(b). The corresponding ferroelectric-to-paraelectric phase transition leading to low-dimensional symmetry, including a centrosymmetric and biaxial orthorhombic (*Fdd*2) and monoclinic (2/*m*) crystal structures and a build-up of crystal structure asymmetry with reference to the optic-axis have been reported^28–32^. The partially broken rotational symmetry is evident from the scheme of fast-axis orientation in the momentum space (via stereographic projection) given in Fig. 1(b), which exhibit different topologies, marked in different colors at different regions. This feature of the field-induced transformation of the crystal structure is found to be highly desirable for the demonstration of simultaneously present SOI and controllable observation of SHEL. The complex topology of the fast-axis orientation (*μ*) perceived by the spread of wavevectors in a paraxial optical beam, as it propagates through a biaxial crystal is given by^33^

2 $$\mu =\frac{1}{2}{\tan }^{-1}(\frac{\sin \,2\varphi \,\cos \,\theta }{{\rm{\Delta }}\,{\sin }^{2}\theta -\,\cos \,2\varphi \,\cos \,\theta })$$

where *θ* is the angle between the wavevector and the *c*-axis of the KDP crystal and *ϕ* is the azimuthal coordinate of the stereographic projection plane (Fig. 1(b)). It is important to note here the electric field *E*_*x*_ dependency of *μ* through the asymmetric term Δ, given by ${\rm{\Delta }}=\sqrt{{n}_{z}^{2}({n}_{y}^{2}-{n}_{x}^{2})/{n}_{x}^{2}({n}_{y}^{2}-{n}_{z}^{2})}$.

To understand the SOI of light in optical crystals, consider circularly polarized (CP) paraxial light beam propagating along *Z*-axis, making an angle *θ*_*c*_ with the optic-axes of the biaxial crystal (Fig. 1(a)). Different local topologies in the fast-axis orientation, shown in Fig. 1(b), leads to different manifestation of the SOI. To exemplify this, we consider the plane wave component $|{\sigma }_{\pm }\rangle$ in the paraxial beam, which makes an angle *θ* with the *z*-axis. The final state, after propagation through the crystal, can be expressed in the right- (R), $|{\sigma }_{+}\rangle$ and left- (L), $|{\sigma }_{-}\rangle$ CP basis as^34^

3 $${E}_{out}=\,\cos (\frac{\delta }{2})|{\sigma }_{\pm }\rangle +i\,\sin (\frac{\delta }{2})|{\sigma }_{\mp }\rangle {e}^{\pm i2\mu }$$

where *δ* is the phase retardation experienced by the linear polarization component of the plane wave due to linear birefringence of the crystal. The second term in eqn. (3) represents the SOI, with ${\sin }^{2}(\delta /2)$ fraction of photons flip its SAM handedness and transform to its orthogonal state, accompanied by the PB phase factor ${e}^{\pm i2\mu }$. As shown in Fig. 1(b), the topological structures identified as *node* (green circle) and *lemon* (red circles) play a significant role in the SOC, and the linear gradient portion (blue lines) contribute to the SHEL, respectively via azimuthally and linearly varying PB phase for a range of plane-waves (*Δk*) in the paraxial beam around the central plane wave. The SOC is typically characterized by extracting the associated helical phase structure either using the Fourier fringe analysis^34^ or wavevector resolved Stokes polarimetry^18^ respectively representing the scalar and vector optics treatment.

**Figure 2:**
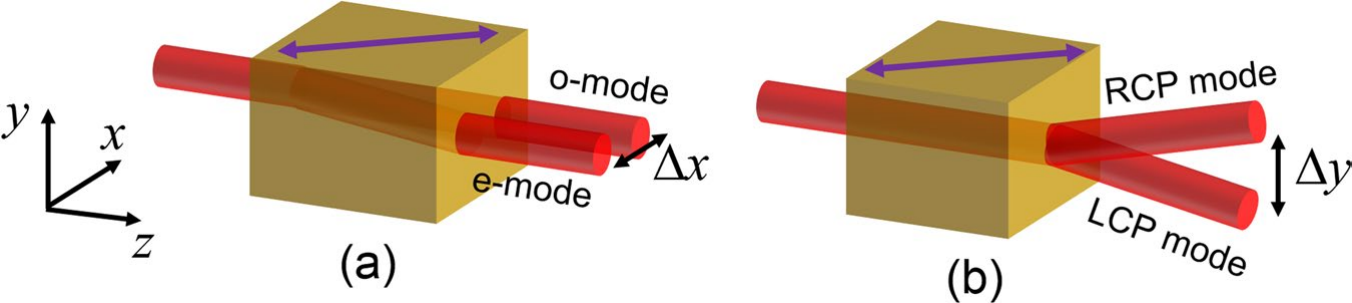
Schematic illustration of the analogy between (**a**) spatial walk-off of *o*-mode and *e*-modes due to linear birefringence and (**b**) SHEL due to induced circular birefringence. Optic-axis of the crystal is indicated by purple color arrow.

However, as stated, due to the complex fast-axis orientation around the symmetry axis (Fig. 1(b)) the weak measurement and Stokes polarimetry techniques are found not suitable to characterize the SHEL in the biaxial crystal, and we use CMMA as an alternate measurement technique. For that, we consider the SHEL, i.e., the spin-dependent energy re-distribution in the transverse direction due to broken symmetry of the crystal system as the induced circular birefringence that gives rise a spatial walk-off of the right and left circular polarization (RCP and LCP) modes linked to the recently reported transverse birefringence^16^, a noteworthy analogue to the spatial walk-off of ordinary and extra-ordinary modes due to the linear birefringence^32^ as schematically illustrated in Fig. 2. In the case of linear birefringence, the additional dynamic phase (retardance) gradient acquired by the linearly polarized extra-ordinary mode (e-mode) in the medium results in an in-plane beam shift with respect to the optic-axis (Fig. 2(a)), whereas the additional Pancharatnam-Berry type geometric phase acquired by the right and left circular polarization modes result in out-of-plane beam shift (Fig. 2(b)) that leads to the SHEL. Thus, a careful investigation of the circular birefringence in a linear birefringent medium provides the glimpses of the SHEL. Accordingly, we characterize the induced circular birefringence in the crystal through the extensive CMMA and introduce the circular anisotropy coefficient (*γ*), as a measure of the SHEL. The fast-axis orientation (*μ*) of the crystal is obtained from the linear anisotropy coefficients (*α* and *β*) using 2*μ* = **arg**(*α* + *iβ*). The anisotropy coefficients *α*, *β* and *γ* with *α*^2^ + *β*^2^ + *γ*^2^ = 1 defines the relative measure of the birefringence of the crystal between horizontal-vertical, diagonal-anti diagonal and right-left circular polarization modes, respectively and can be extracted directly from the MM elements^35^.

**Figure 3:**
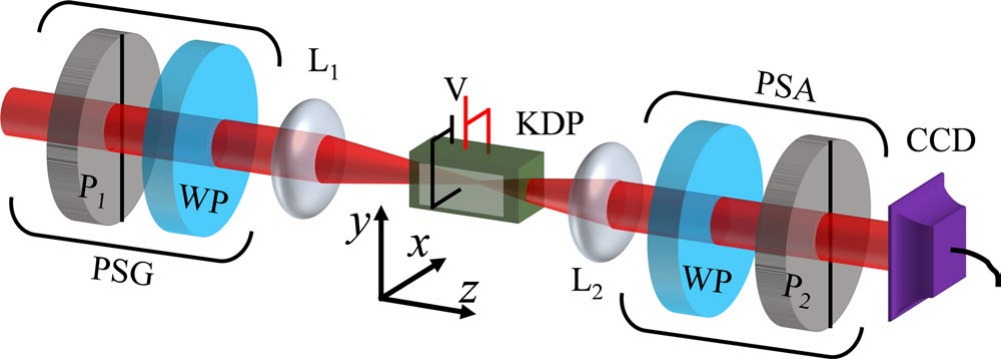
Experimental scheme for the measurement of conoscopic Mueller matrix elements.

The conoscopic MM elements of the KDP crystal under different applied electric field are measured using the experimental setup schematically shown in Fig. 3. A He-Ne laser beam (*λ* = 632.8 nm) is focused into the *c*-cut KDP crystal (*l* × *d* = 4 cm × 1 cm) using a lens L_1_ (focal length f_1_ = 2 cm) and collimated back using lens L_2_ (f_2_ = 3 cm). The biaxial phases of the KDP crystal are obtained by applying electric field *E*_*x*_ using a DC voltage source (V) connected across the crystal as shown in the figure, where ${E}_{x}={\rm{V}}/d={\rm{V}}\times {10}^{2}$. A polarization state generator (PSG), made up of a vertical-oriented Glan-Thompson polarizer (P_1_) and a zero-order half- or quarter- wave plate (WP), preselect the beam in linear or circular states of polarization (SoP) and a polarization state analyzer (PSA) post-select the output beam on different linear and circular polarization states and the intensity images are recorded using a CCD camera (Spiricon, USA) connected to a computer. The method of extraction of MM elements and anisotropy coefficients from the recorded intensity images are detailed in the Method section.

## Results

**Figure 4:**
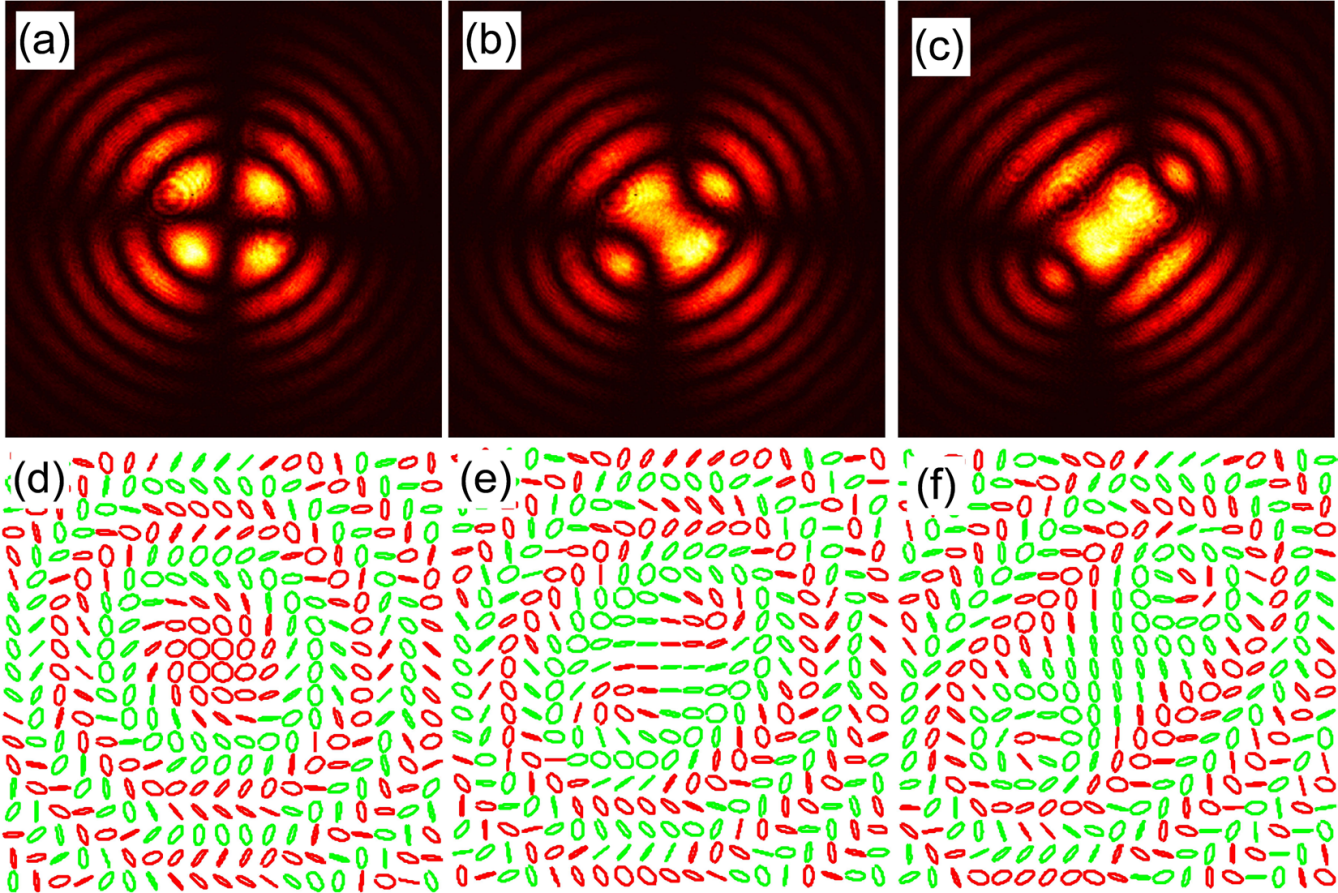
Conoscopic interference pattern (first row) and the polarization ellipse map (second row) of the output beam for different applied fields, ${E}_{x}=0\,{\rm{V}}/m$ (**a,d**), ${E}_{x}={\rm{0.7}}\times {\rm{1}}{0}^{2}\,{\rm{V}}/m$ (**b,e**) and ${E}_{x}={\rm{2}}\times {\rm{1}}{0}^{2}\,{\rm{V}}/m$ (**c,f**).

The transformation of the KDP crystal is characterized first by mapping the well-known conoscopic interference pattern^36^ under the electric fields ${E}_{x}=0\,{\rm{V}}/m$, ${E}_{x}={\rm{0.7}}\times {\rm{1}}{0}^{2}\,{\rm{V}}/m$ and ${E}_{x}={\rm{2}}\times {\rm{1}}{0}^{2}\,{\rm{V}}/m$, and are respectively shown in Fig. 4(a,b and c). The rotationally symmetric conoscopic pattern consisting of entangled *isochromate* rings and *isogyre* cross pattern confirm the uniaxial phase of the KDP crystal at ${E}_{x}=0\,{\rm{V}}/m$^34^ (Fig. 4(a)), while the broken symmetry of the pattern under the applied electric field shows the weak and strong biaxial phases of the crystal (Fig. 4(b,c)). Next, we send right CP Gaussian beam along the c-axis of the crystal and the polarimetric measurements of the output beam further confirms the uniaxial and the biaxial phases of the crystal. The polarization map of the beam exhibits a rotationally symmetric spiral topology around the C-point^37^ as shown in Fig. 4(d). This polarization topology arises from the superposition of right CP Gaussian beam with doubly charged left CP Laguerre-Gaussian (LG) beam of light propagating through the uniaxial crystal. Figure 4(e) and (f) exhibit a broken symmetry with two well-separated lemon topologies around the two optic-axes of the biaxial KDP crystal, and is due to the local superposition of right CP Gaussian beam with single-charge of left CP LG beam.

**Figure 5:**
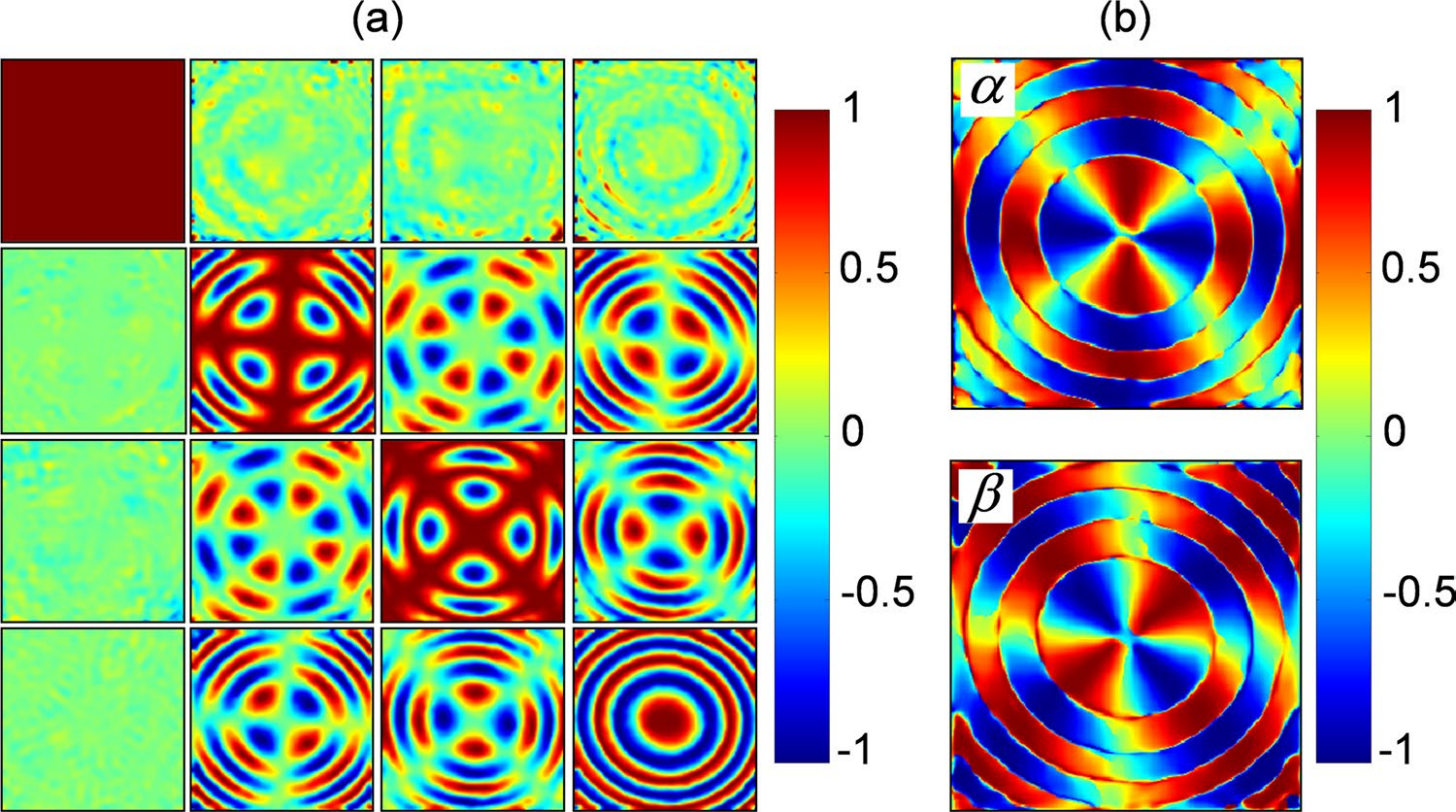
(**a**) conoscopic Muller matrix elements, and (**b**) linear anisotropy coefficients (*α, β*) of the KDP crystal at uniaxial phase, for ${E}_{x}=0\,{\rm{V}}/m$.

The experimentally obtained MM elements of the KDP crystal in uniaxial phase (for ${E}_{x}=0\,{\rm{V}}/m$) are given in Fig. 5(a). The 4 × 4 MM elements are normalized with respect to the first element *M*_*00*_ that represents the total intensity of light passing through the crystal, which is set to unity. The elements in the first column (*M*_10_, *M*_20_ and *M*_30_) and first row (*M*_01_, *M*_02_ and *M*_03_) represent the polarizance and dichroism of the crystal for different paraxial beam wave vector directions passing through the crystal. The values are almost zero close to the optic-axis implying that the KDP crystal does not possess such characteristics to start with. However, these elements exhibit non-negligible values away from the optic-axis, especially near the periphery, due to the attenuation of obliquely incident rays, possibly due to reflection loss at the crystal interfaces. The remaining elements of the MM construct a 3 × 3 sub-matrix, representing the linear and circular birefringence properties of the crystal, from which the linear (*α* and *β*) and circular anisotropy coefficients (*γ*) are calculated as described in the method section^35^. The *α* and *β* behavior given in Fig. 4(b) reflect the rotational symmetry of the system and the isogyre-like cross pattern. Important to note here the sign flip in the radial direction due to singular jumps which occur at π multiples of the crystal retardance, and is taken into consideration in the calculation of fast axis orientation.

**Figure 6:**
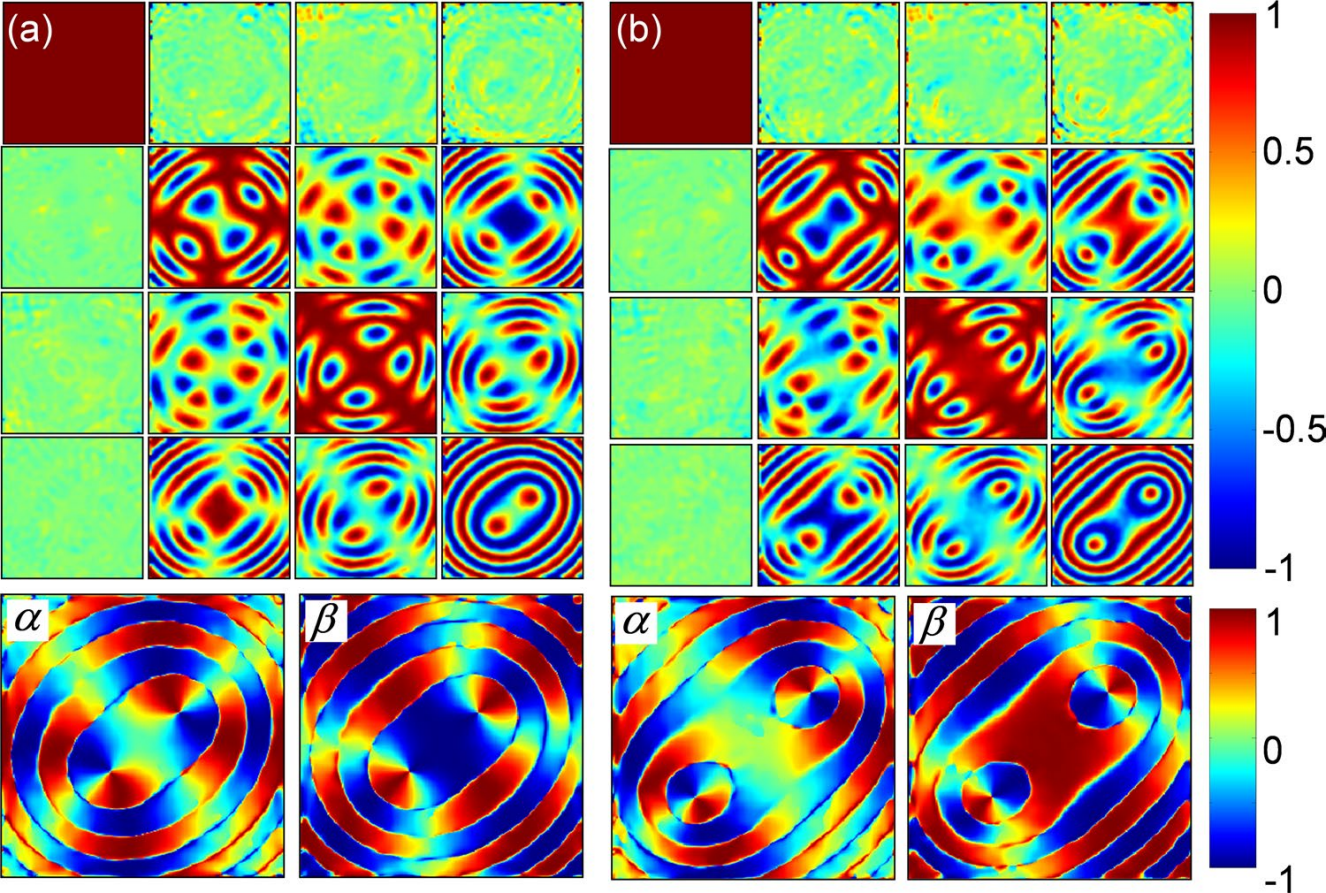
(**a**), (**b**) conoscopic Muller matrix elements (first row), and linear anisotropy coefficients (*α, β*) (second row) of the KDP crystal in orthorhombic and monoclinic biaxial crystal phases respectively for (**a**) ${E}_{x}={\rm{0.7}}\times {\rm{1}}{0}^{2}\,{\rm{V}}/m$ and (**b**) ${E}_{x}={\rm{2}}\times {\rm{1}}{0}^{2}\,{\rm{V}}/m$.

However, the simple situation changes dramatically with the application of electric field (*E*_*x*_) across the KDP crystal – transforming from the rotationally symmetric uniaxial to asymmetric biaxial crystal phase. The paraboloidal form retardance of the uniaxial KDP crystal gets modified significantly, becoming broader and shallower double well type with the application of *E*_*x*_^34^. We report here two significant points in this continuous transformation, corresponding to the fields ${E}_{x}={\rm{0.7}}\times {\rm{1}}{0}^{2}\,{\rm{V}}/m$ and ${E}_{x}={\rm{2}}\times {\rm{1}}{0}^{2}\,{\rm{V}}/m$ applied across the crystal, which correspond to the appearance of weak and strong asymmetry respectively. The experimentally obtained MM elements, given in Fig. 6(a) and (b) clearly indicate the appearance of biaxiality in the KDP crystal, a hitherto less known and investigated crystal structure to be reported at room temperature^38,39^. We identify from the MM elements that the KDP crystal for ${E}_{x}={\rm{0.7}}\times {\rm{1}}{0}^{2}\,{\rm{V}}/m$ and ${E}_{x}={\rm{2}}\times {\rm{1}}{0}^{2}\,{\rm{V}}/m$ respectively belong to the orthorhombic and monoclinic biaxial crystal structures of lower symmetry^40^. Accordingly, we infer that the crystal structure of the transparent KDP crystal, due to the applied electric field undergoes a continuous transformation from uniaxial tetragonal (point group $\bar{4}2m$) to biaxial orthorhombic (*Fdd*2) and monoclinic (2/*m*) structure of decreasing symmetry^28,40^. This transformation offers a unique opportunity to explore the changes in the optical properties of the crystal at room temperature, leading to a complete understanding of the SOI in optical crystals. The extracted linear anisotropy coefficients (*α* and *β*) of the two biaxial phases of the crystal are shown in the second row of Fig. 6.

**Figure 7:**
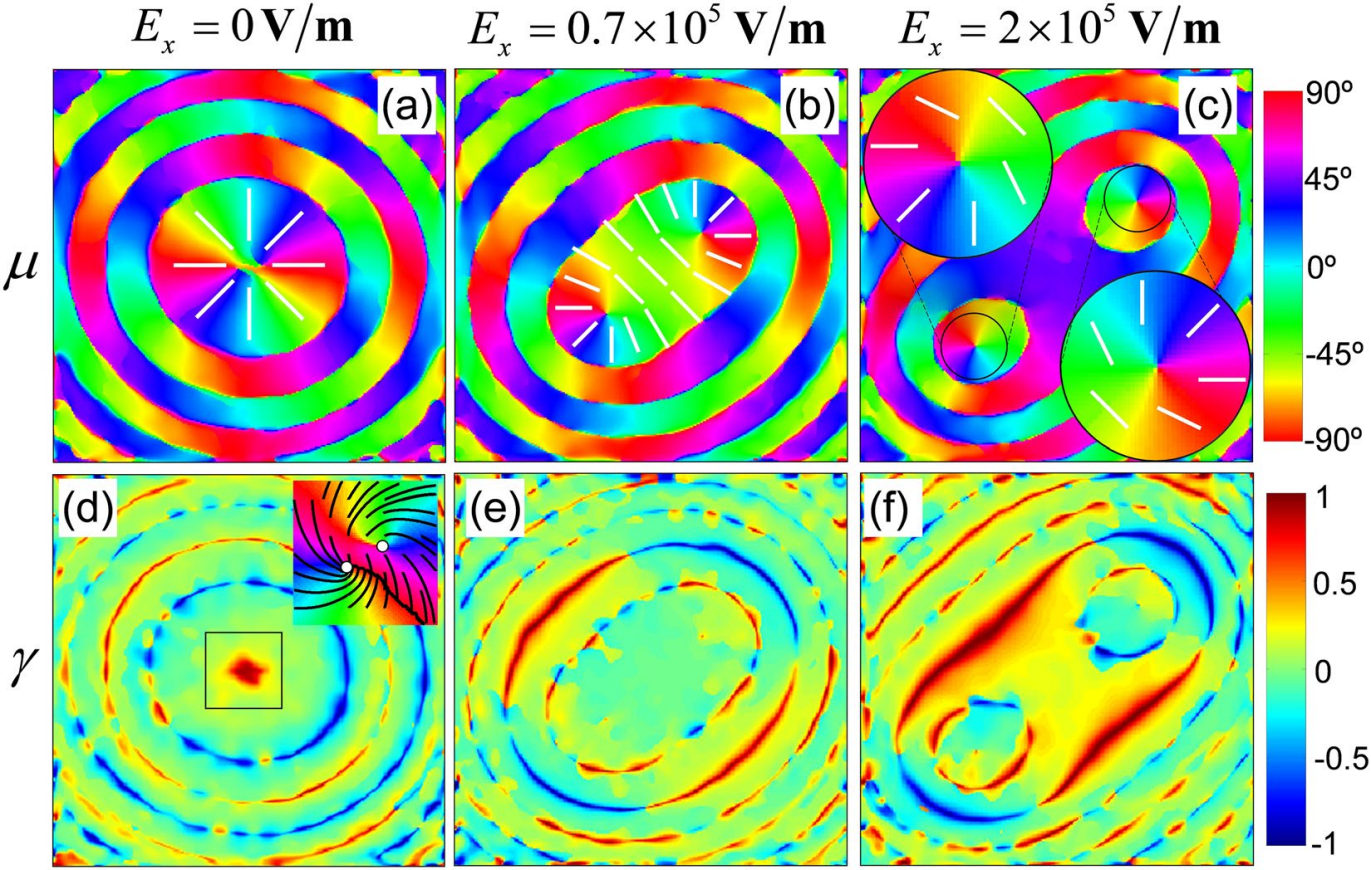
Circular anisotropy coefficient (*γ*) for (**a**) ${E}_{x}=0\,{\rm{V}}/{\rm{m}}$, (**b**) ${E}_{x}=0.7\times {10}^{5}\,{\rm{V}}/{\rm{m}}$ and ${E}_{x}=2\times {10}^{5}\,{\rm{V}}/{\rm{m}}$ applied across the biaxial crystal.

The topological transformation of fast-axis orientation and the non-vanishing circular anisotropy coefficient of the KDP crystal under strong electric field are the central results of our experiment. The fast-axis orientation (*μ*) exhibits rotationally symmetric node topology with *μ* = *ϕ* as shown in Fig. 7(a) for ${E}_{x}=0\,{\rm{V}}/m$, corresponding to the uniaxial phase of the crystal. When the crystal symmetry, with reference to the optic-axis and the direction of propagation, is broken due to the application of transverse electric field, the node topology splits into two lemon structures with $2\mu =\varphi ^{\prime}$ and pushes away from the center, the consequence of uniaxial-to-biaxial transformation of the KDP crystal. Here $\varphi ^{\prime}$ is the local azimuthal coordinate around the newly formed optic-axes. Figure 7(b) and (c) show the slightly and well separated lemon structures corresponding to the weak and strong biaxiality of the crystal in response to the applied field.

Significantly, the appearance of non-zero circular anisotropy coefficient is quite surprising for the traditional crystal optics, since it directly indicates the presence of circular birefringence in a linear birefringent medium due to the SHEL. However, the unexpected presence of *γ* in the uniaxial phase of the crystal, as shown in the beam center in Fig. 7(d), is due to the inherent, weak biaxiality of the KDP crystal used in the experiment. This can be seen in the zoomed inset, where the white circles are the optical axes surrounded by *lemon*-type polarization ellipse stream lines^37^. In addition, the variations in *γ* appears as concentric circles at every * π* phase variation as mentioned previously. In addition to these, the appearance of strong circular anisotropy coefficient is significant for the biaxial phases of the KDP crystal as shown in Fig. 7(e) and (f). The cross-shaped *γ* behavior gets enhanced, and moves from the periphery to the center as the crystal asymmetry increases with the applied field. In the region closer to the two optic-axes *γ* is almost negligible, due to the local rotational symmetry. These results are considered as strong evidence for the presence of circular birefringence in the biaxial crystal and hence the SHEL, in response to the symmetry-breaking due to the applied electric field. An overall tilt of 45° in the pattern may be attributed to the electro-gyration effect^38,41^ arising due to the field induced crystal asymmetry and will be detailed elsewhere.

## Discussion

The SOI of light in a birefringent crystal is governed mainly by two parameters: the retardance (*δ*) and the fast-axis orientation (*μ*), that respectively decide the efficiency and the nature of the associated OAM transfer. The wave-vector resolved CMMA of the KDP crystal characterizes the SOI by extracting the key parameters, *δ* and *μ* from the linear anisotropy coefficients. It is important to note here the average efficiency of the SOI for a paraxial light beam propagating along the c-axis of the crystal is limited to 50% by the factor $\int {\sin }^{2}(\delta /2)\le 0.5$ from eqn. (3)^34^. We thus focus here on the fast-axis orientation and the associated nature of SOI of light with the crystal.

As seen from Fig. 6(a), *μ* exhibits a rotationally symmetric node topology that results in the generation of doubly charged vortex beam with IOAM of $l=+{\bf{2}}\hslash$ per photon. This is a well-studied manifestation of SOI in terms of SOC in uniaxial crystal^34,42^. This conversion can be understood from the angular momentum (AM) conservation point of view: let us consider a photon in right circularly polarized Gaussian beam, carrying an intrinsic OAM of $l={\bf{0}}\hslash$ and SAM $\sigma ={\bf{1}}\hslash$ giving rise to a total AM (TAM), $J=\sigma +l={\bf{1}}\hslash$. Upon interacting with the crystal, the photon SAM switches to $\sigma =-{\bf{1}}\hslash$ due to flipping in the SAM handedness. The rotational symmetry of the system provides conservation of TAM and the change in TAM by a factor ${\bf{2}}\hslash$ due to $\Delta \sigma =-{\bf{2}}\hslash$ which is completely transformed to the intrinsic OAM with $l=+{\bf{2}}\hslash$. The efficiency of this process is only half and accordingly, half of the photons in the beam acquire an intrinsic OAM, resulting in the LG mode with charge $l=+{\bf{2}}\hslash$. However, the application of strong electric field across the crystal breaks the rotational symmetry of *μ* and completely changes the nature of SOI. The complex and asymmetric structure of *μ* mainly involves three local topologies as mentioned previously, a node at the periphery, two lemons around the optic-axes and a linear gradient at intermediate regions (Fig. 1(b)) which are experimentally verified in Fig. 7(b) and (c). Photons propagating around the periphery undergo SOC as discussed above. Whereas, the photons propagating through the lemon structure around the ‘newly formed’ optic-axes acquire intrinsic OAM of $l=+{\bf{1}}\hslash$, and results in the LG mode with a unit charge. It was shown that these observations alone do not lead to the TAM conservation^18,43,44^ of the beam-field due to the absence of continuous rotational symmetry, and the remaining ${\bf{1}}\hslash$ may be transferred to the medium. The two SOIs mentioned above, are very similar to the SOCs of the q-plate, sub-wavelength dielectric and plasmonic metasurfaces with index $q=1$ and $q=1/2$ respectively^3–5^.

Significantly, the linear gradient in the fast-axis orientation (blue lines in Fig. 1(b)), is not associated with the any of the azimuthal coordinate, and photons passing through this region do not acquire intrinsic OAM and do not involve in the either of the SOCs. On the other hand, these photons acquire an additional extrinsic OAM accounted to the linearly varying accumulation of PB phase, and results in SHEL^8,9,16^. The subsequent spin dependent shift, the spatial walk-off of circular polarization modes resulting in an induced circular birefringence and leads to the non-zero circular anisotropy coefficient (*γ*) at regions where the symmetry is maximally broken as shown in Fig. 7(e) and (f). These original observations are quite surprising for traditional crystal optics since it implies the appearance of weak circular birefringence in biaxial KDP crystal. Similar kind of results have been demonstrated recently in quartz wave plate as the manifestation of SHEL by our group using the weak measurement technique^16^. It is important to note here the crucial difference of the present work part the measurement technique point of view. In addition to the asymmetry in the wave plate was brought by tilting its optic-axis with respect to the propagation direction, whereas, here the symmetry is broken by the applied electric field by means of electro- and piezo-optic effects, which provides a fine tuning of the SHEL.

This remarkable observation of the conversion of the SAM to extrinsic OAM completes the AM conservation law in biaxial crystals by the definition, TAM = SAM + intrinsic OAM + extrinsic OAM, and addresses directly the recently reported non-conservation of AM and the generation of higher-order OAM side bands^18,43,44^ in biaxial crystals. Accordingly, a photon entering a biaxial crystal at an arbitrary angle can have the following possibilities: photon flips its SAM handedness and acquires an intrinsic AM of ${\rm{2}}\hslash$ or acquire an intrinsic AM of ${\rm{1}}\hslash$, or acquire an extrinsic AM and deviate from the propagation path, with a note that the maximum possibility to flip the handedness is only half and the unbalanced AM in the latter scenario may be transferred to the crystal. On the other hand, the same photon exhibits a 50% of probability to pass through the crystal without the spin flip.

## Method

A Mueller matrix (MM) represents an optical system that transforms the Stokes vector as given below^45^,

4 $${E}_{out}=(\begin{array}{c}{S^{\prime} }_{0}\\ {S^{\prime} }_{1}\\ {S^{\prime} }_{2}\\ {S^{\prime} }_{3}\end{array})=(\begin{array}{cccc}{M}_{00} & {M}_{01} & {M}_{02} & {M}_{03}\\ {M}_{10} & {M}_{11} & {M}_{12} & {M}_{13}\\ {M}_{20} & {M}_{21} & {M}_{22} & {M}_{23}\\ {M}_{30} & {M}_{31} & {M}_{32} & {M}_{33}\end{array})(\begin{array}{c}{S}_{0}\\ {S}_{1}\\ {S}_{2}\\ {S}_{3}\end{array})$$

where *S*_*j*_ and ${S^{\prime} }_{i}$ are the Stokes vectors of input and output field, and *M*_*ij*_ are the 4 × 4 MM elements, with *i*, *j* = 0−3. The elements *M*_*i*0_ and ${M}_{0j}$ characterize the polarizance and dichroism property that determined by the imaginary part of the anisotropy of the medium. For a non-depolarizing and non-absorbing medium, the birefringence properties are described by the remaining elements, where the linear birefringence of the medium is represented by,

5 $${M}_{L}=(\begin{array}{cccc}1 & 0 & 0 & 0\\ 0 & 1 & 0 & 0\\ 0 & 0 & {M}_{22} & {M}_{23}\\ 0 & 0 & {M}_{32} & {M}_{33}\end{array});{M^{\prime} }_{L}=(\begin{array}{cccc}1 & 0 & 0 & 0\\ 0 & {M}_{11} & 0 & {M}_{13}\\ 0 & 0 & 1 & 0\\ 0 & {M}_{31} & 0 & {M}_{33}\end{array})$$

here *M*_*L*_ and ${M}_{L}^{\prime}$ are respectively represent the horizontal - vertical and diagonal - anti-diagonal birefringence MM of the medium. Correspondingly, the circular birefringence is represented by,

6 $${M}_{C}=(\begin{array}{cccc}1 & 0 & 0 & 0\\ 0 & {M}_{11} & {M}_{12} & 0\\ 0 & {M}_{21} & {M}_{22} & 0\\ 0 & 0 & 0 & 1\end{array})$$

Importantly, the linear anisotropic coefficients and the circular anisotropic coefficient *γ* are determined by the difference between the off-diagonal elements of *M*_*L*_, ${M}_{L}^{\prime}$ and *M*_*C*_ respectively^35^. Accordingly, the fast axis orientations of the crystal are directly related to the conoscopic MM elements *M*_23_, *M*_32_, *M*_13_ and *M*_31_, while the elements *M*_12_ and *M*_21_ reflect the SHEL in the crystal.

Experimentally, the MM elements are obtained using Stokes polarimetry measurements using the following relations^46^,

7 $${\begin{array}{cc}{M}_{i0}=\frac{{S}_{i}^{\prime} ({\bf{V}})+{S}_{i}^{\prime} ({\bf{H}})}{2} & {M}_{i1}=\frac{{S}_{i}^{\prime} ({\bf{V}})-{S}_{i}^{\prime} ({\bf{H}})}{2}\\ {M}_{i2}=\frac{{S}_{i}^{\prime} ({\bf{D}})-{S}_{i}^{\prime} ({\bf{A}})}{2} & {M}_{i3}=\frac{{S}_{i}^{\prime} ({\bf{R}})-{S}_{i}^{\prime} ({\bf{L}})}{2}\end{array})}_{i=0,1,2,3}$$

here ${S}_{i}^{\prime} ({\bf{X}})$ are the four Stokes vector elements of the output field for a pre-selected input field in the state of polarization (SoP) **X**, which stands for **H, V, D, A, R** and **L** respectively the Horizontal, Vertical, Diagonal, Anti-diagonal, Right-circular and Left-circular SoP. The Stokes vectors for the input SoP can be obtained from the intensity measurement of different polarization projections from the relations^45^,

8 $$\begin{array}{cc}{S}_{0}^{\prime} ({\bf{X}})={\bf{H}}({\bf{X}})+{\bf{V}}({\bf{X}}) & {S}_{1}^{\prime} ({\bf{X}})={\bf{H}}({\bf{X}})-{\bf{V}}({\bf{X}})\\ {S}_{2}^{\prime} ({\bf{X}})={\bf{D}}({\bf{X}})-{\bf{A}}({\bf{X}}) & {S}_{3}^{\prime} ({\bf{X}})={\bf{R}}({\bf{X}})-{\bf{L}}({\bf{X}})\end{array}$$

**Figure 8:**
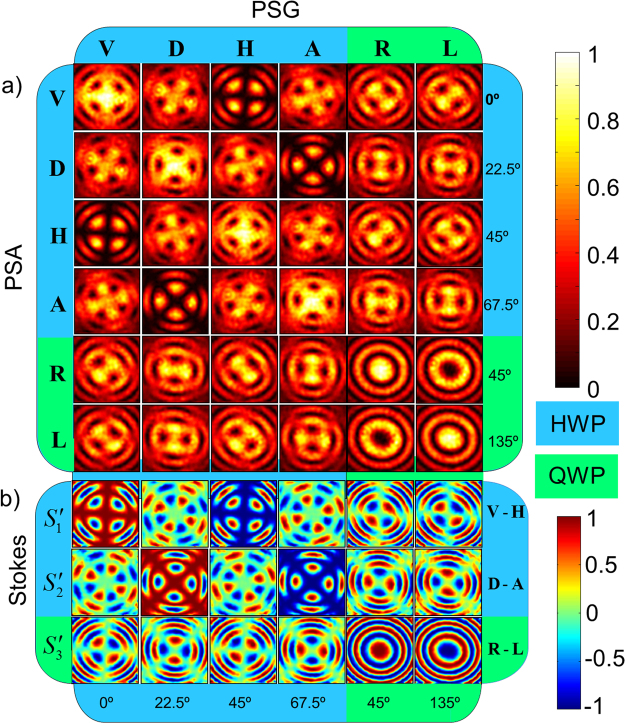
(**a**) Recorded intensity images corresponding to 36 polarization projections of KDP crystal in uniaxial phase, (**b**) The Stokes parameters calculated from the projection measurements.

The pre-selection of different SoP, necessary to extract the Mueller matrix elements, is achieved using the polarization state generator (PSG) in the experimental set up shown in Fig. 3. The **V, D, H** and **A** SoP are generated with the HWP oriented at 0°, 22.5°, 45° and 67.5° respectively with respect to the *y*-axis, and the R and L states are pre-selected by carefully replacing the HWP with a QWP which is orientated at 45° and 135°, respectively. After the interaction of the pre-selected paraxial field with the crystal, the output beam is post-selected onto the six (**H, V, D, A, R** and **L**) SoP using the polarization state analyzer (PSA) and the resulting intensity images are recorded using the CCD camera. Calculation of the Stokes parameters using Eqn. 8 is carried out after digitally smoothening the recorded images using Fourier fringe analysis to remove the high-frequency patterns in the images, which appear possibly due to dust and scattering from the optical components. The recorded intensity images and the calculated Stokes parameters are shown in Fig. 7(a) and (b), respectively. Columns and rows in the Fig. 8 are corresponding to the pre-selections and the post-selections respectively. The first four columns and rows represent the pre- and post-selection on to the linear SoP obtained by rotating the HWP, and the last two are on to the circular SoP obtained using the QWP. The angles given in the figure correspond to the orientation of the wave plates in the PSG and PSA. Accordingly, these 36 projection images which represent the complete birefringence properties of the crystal is then reduced to 18 Stokes parameters using the Eqn. 8, and to 16 MM elements using the Eqn. 7, and finally reduced to two linear and circular anisotropy coefficients *α*, *β* and *γ* by following the calculations described in ref.^35^.
